# Usefulness of heart rate variability indices in assessing the risk of an unsuccessful return to work after sick leave in depressed patients

**DOI:** 10.1002/npr2.12121

**Published:** 2020-07-05

**Authors:** Toshikazu Shinba, Keizo Murotsu, Yosuke Usui, Yoshinori Andow, Hiroshi Terada, Mami Takahashi, Rie Takii, Michiko Urita, Satoshi Sakuragawa, Miwa Mochizuki, Nobutoshi Kariya, Saori Matsuda, Yusuke Obara, Hanae Matsuda, Yoshitaka Tatebayashi, Yoshiki Matsuda, Go Mugishima, Takaki Nedachi, Guanghao Sun, Tomoko Inoue, Takemi Matsui

**Affiliations:** ^1^ Department of Psychiatry Shizuoka Saiseikai General Hospital Shizuoka Japan; ^2^ Department of Psychiatry Shizuoka Red Cross Hospital Shizuoka Japan; ^3^ Maynds Tower Mental Clinic Tokyo Japan; ^4^ Affective Disorders Research Project Tokyo Metropolitan Institute of Medical Science Tokyo Japan; ^5^ Hakuju Institute for Health Science Tokyo Japan; ^6^ Graduate School of System Design Tokyo Metropolitan University Hachioji Japan; ^7^ Yoga Mental Clinic Tokyo Japan; ^8^ Aoi Clinic Shizuoka Japan; ^9^ Industrial Research Institute of Shizuoka Prefecture Shizuoka Japan; ^10^ Yonenomiya Clinic Fuji Japan; ^11^ Department of Psychology, School of Human and Social Sciences Fukuoka Prefectural University Fukuoka Japan; ^12^ Graduate School of Informatics and Engineering The University of Electro‐Communications Chofu Japan

**Keywords:** autonomic dysregulation, depression, heart rate variability, leave of absence, outcome of reinstatement

## Abstract

**Aim:**

The present study aimed to examine whether heart rate variability (HRV) indices in depressed patients measured at return to work after sick leave are related to the outcome of reinstatement.

**Methods:**

This study included 30 workers who took a leave of absence due to major depressive disorder. HRV was measured twice, once when participants left work and another when they returned to work. One month after returning to work, 19 participants continued their original work (successful return group), while 11 failed to perform their original work (unsuccessful return group). HRV indices including high‐ and low‐frequency components (HF and LF) were calculated in three conditions within a session lasting for about 5 minutes, initial rest (Rest), mental task (Task), and rest after task (After), and were compared between the two participant groups. Psychological states were evaluated using Self‐rating Depression Scale and State‐Trait Anxiety Inventory.

**Results:**

No significant differences were observed in the HRV indices on leaving work between groups. On returning to work, the “unsuccessful return group” exhibited lower HF Rest score, higher HF Task/Rest ratio, and higher LF/HF Rest score than the “successful return group.” Psychological scores improved in both groups.

**Conclusion:**

These results indicate that autonomic dysregulations revealed by HRV measurement at return to work after a leave of absence in MDD patients were related to the outcome of reinstatement and can serve as useful information for the assessment of the risk of unsuccessful return.

## Abstract 2

Autonomic dysregulations revealed by heart rate variability measurement at return to work after sick leave in depressed patients were related to the outcome of reinstatement and can serve as useful information for the assessment of the risk of unsuccessful return.

## Graphical abstract

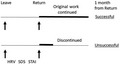

## 1 INTRODUCTION

In the treatment of depression, restoration of social functioning is important in addition to symptomatic recovery and has been used as an outcome measure in clinical evaluation.^1^ Among the various aspects of social functioning in depressed patients, return to work after sick leave has been considered as a significant factor^2^ because it directly impacts the patients’ life and the functioning of the companies that employ them. Previous epidemiological studies indicate that the rate of recurrent absence due to depression is high, and that the second period of sick leave is longer than the first one.^3^, ^4^ Careful management of employees on their return to work is required, because inadequate returns would lead to worsening of their future social functioning.

To facilitate adequate return to work, the prognostic factors related to successful and unsuccessful return need to be determined. A recent review^5^ showed that older age, somatic comorbidity, psychiatric comorbidity, and more severe depression are associated with a lower rate of successful return, while personality trait conscientiousness is associated with higher rate of successful return. In reference to these factors, pre‐return social and occupational training is effective for the sick leave patients, where training elements include simulation of work and its environment.^6^

In search for more objective indices to promote the adequate return, biological parameters were evaluated. Ikeda et al^7^ have reported that prefrontal blood flow was disturbed in depressive patients despite clinical remission and preparation for return. Biological evaluation of residual dysfunction at the time of return will be useful to avoid repeated sickness leaves and to help patients to maintain their daily activities.

To biologically evaluate the residual dysfunction, the present study employed autonomic indices and examined whether these indices are useful in predicting the outcome of the return to work. Heart rate variability (HRV) was used to analyze the autonomic activity. Previous studies have indicated that HRV measurement is useful for evaluating the pathophysiological state of patients with major depressive disorder (MDD).^8^, ^9^, ^10^ In these studies, the power spectrum of heart beat interval data was analyzed both at rest and during a mental task, and several autonomic dysfunctions related to MDD were observed. In particular, the data obtained during the performance of tasks were interesting as it can be used to assess the participant’s ability to perform work. In addition, reactivity to task load will help assess the participant’s autonomic controllability when they are faced with stressful events at work.

In the present study, HRV was measured twice in participants diagnosed with MDD, once when they left work, and another when they returned to work. The relationship between HRV data and the outcome of the return was assessed.

## 2 METHODS

### 2.1 Participants

This study included 30 patients (age: 42.7 ± 12.1 years old, mean ± SD, 16 men and 14 women) diagnosed with MDD based on the Diagnostic and Statistical Manual of Mental Disorders, Fifth Edition (DSM‐5) criteria^11^ and were treated at Shizuoka Saiseikai General Hospital, Shizuoka Red Cross Hospital, Yoga Mental Clinic and Aoi Clinic. All participants provided a written informed consent. The protocol of the study was approved by the Institutional Review Board of Shizuoka Saiseikai General Hospital.

The participants with first‐onset MDD were included in the present study. Those with heart, lung, brain, or neuronal diseases, or other psychiatric diseases, including anxiety disorder, schizophrenia, adjustment disorder, and neurodevelopmental disorder along with those with previous depressive episodes were excluded.

The following participants were enrolled in this study in consecutive order: workers, those who took sick leaves due to MDD, and those who tried to return to their original work after their attending psychiatrist (TS, YU, YA, and HT) acknowledged the remission of MDD symptoms based on DSM‐5 criteria.^11^ During the sick leaves, the psychiatrists treated the participants conventionally with antidepressant medication and supportive psychotherapy. The antidepressants were selected by the attending psychiatrists.

The participants who underwent pre‐return social and occupational training before the start of the original work were excluded because the training could have influenced the outcome of their return. The participants who changed the content of work when they returned to work were also excluded, because the change of work could affect the outcome of reinstatement.

### 2.2 Study design

**Figure 1:**
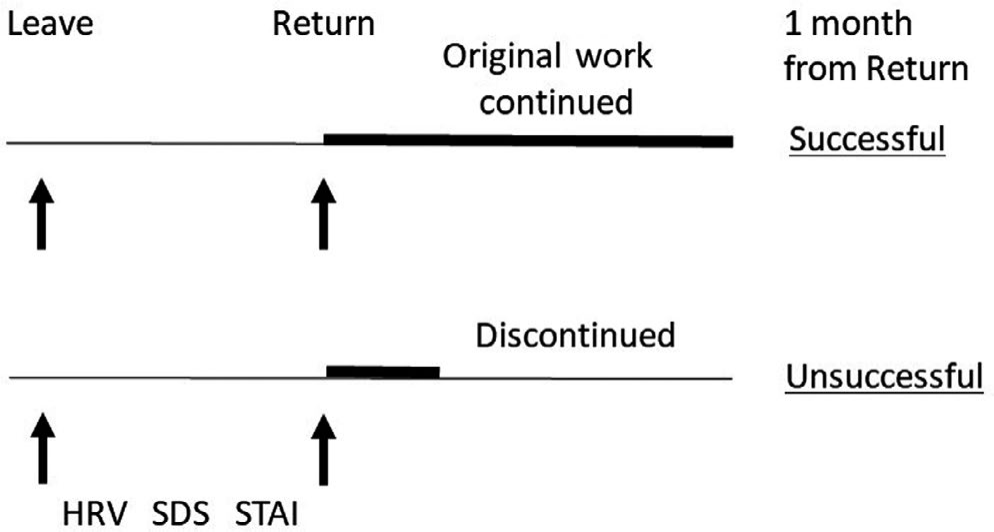
The protocol of heart rate variability (HRV) measurement in successful return and unsuccessful return groups. HRV indices together with Self‐rating Depression Scale (SDS) and State‐Trait Anxiety Inventory (STAI) scores were measured at leave from work and at return. Continuation of original work is designated by thick horizontal lines. Outcome of reinstatement was evaluated as successful or unsuccessful at 1 month following the return to work.

HRV indices were measured twice, once when the participants left work and once when they returned to work (Figure 1). Psychological states were evaluated at the same time using Self‐rating Depression Scale (SDS)^12^ and state score of State‐Trait Anxiety Inventory (STAI‐state).^13^

### 2.3 Successful and unsuccessful return groups

One month following their return to work, the participants were divided into two groups depending on their state of work (Figure 1). “Successful return (SR) group” comprised participants who maintained their original work. “Unsuccessful return (UR) group” comprised participants who discontinued the original work by changing the content of work after return, taking another sick leave, or resigning from the work. The work conditions were verified using the information obtained from the affiliated company.

Evaluation of work condition at a longer interval from the return should be informative to practically judge the return outcome. However, various occupational, social, and personal events affecting the return outcome could arise if a longer interval between the return and the work condition evaluation was employed, and the analysis on the significance of autonomic data at the return for the outcome would be more complex. Thus, in the present study, 1 month was set for evaluation interval to more directly assess the effect of autonomic data at the return.

### 2.4 HRV measurement

At least 5 minutes of adaptation period was introduced before the start of the measurement. During the measurement, the participant was seated on a chair with the electrocardiogram (ECG) electrodes of a wireless amplifier (RF‐ECG, GM3, Tokyo, Japan) attached to the chest. ECG was recorded conventionally with a gain of 10 000 and time constant of 0.1 seconds, and the data were stored in a computer with a sampling frequency of 200 Hz. R peaks were used to make the R‐R interval trend data. R‐R intervals between the range of 273 and 1500 milliseconds were used for analysis to exclude paroxysmal heart beats. When a R‐R interval was omitted, it was replaced by the average of the preceding and following intervals. The R‐R interval trend data were resampled using the mean heart rate (HR), and their fluctuation was analyzed using the maximum entropy method (MemCal, GMS, Tokyo, Japan) to determine the low‐frequency (LF) and high‐frequency (HF) components of the spectrum every 2 seconds by integrating the power to corresponding frequency intervals (0.04‐0.15 Hz for LF; 0.15‐0.4 Hz for HF)^14^ for the preceding 30‐seconds period. R‐R intervals were also converted to HR (/min). The maximum entropy method was selected for the power spectrum analysis because it has been successfully applied to trend data with a minimum duration of 30 seconds and is useful for studies incorporating measurements of multiple behavioral states.^15^

Previous studies have indicated that LF is related to both sympathetic and parasympathetic activities, and reflects baroreceptor‐related blood pressure regulation.^16^ HF reflects parasympathetic activity related to respiratory frequency.^17^ Respiration was monitored, and its frequency was confirmed to be within the range of 0.15‐0.4 Hz in each participant.^18^, ^19^ When the respiratory frequency exceeded the aforementioned range, the participants were asked to modulate the rate of breathing and then the measurement was repeated.

### 2.5 Experimental protocol

ECG was recorded in three different conditions. First, the participants were instructed to relax as much as possible on the chair for approximately 60 seconds (initial rest; Rest). Then, the participants were engaged in a random number generation task^20^ for 100 seconds (mental task; Task). After the task, ECG was recorded for another 60 seconds while the participant was in a relaxed state (rest after task; After).

HF, LF, LF/HF, and HR were averaged in the interval from 30 seconds after the onset to the end of each condition to exclude any data at the beginning of each new period that may still reflect the previous condition.^9^, ^10^ The data in Task and After conditions were expressed as ratios to the data in Rest condition (ie, the Task/Rest ratio and the After/Rest ratio).

In the random number generation task, the participants were instructed to orally generate a random series of 100 digits using the numbers 0 through 9 at the rate of 1 Hz. The generation rate was indicated by a metronome click sound. They were requested to concentrate on this task as much as possible. To evaluate randomness in the generated digit series, counting bias (CB; frequency of counting‐up or counting‐down), interval bias (IB; frequency of same inter‐digit intervals), and random number generation index (RNG; frequency of same digit pairs) were calculated according to our previous study.^20^

### 2.6 Statistics

The differences between the HRV, HR, SDS, STAI, and randomness data at leave from work and at return to work in all participants were examined using paired t‐test. The differences in age and duration of sick leave between the SR group and UR group were evaluated using t test. The differences in HR and HRV data between the two groups were evaluated using two‐way analysis of variance with post hoc Bonferroni test, and the effects of time (leave vs. return), reinstatement outcome (SR vs. UR), and interaction were analyzed (Prism 5, Graph Pad). The distribution of male and female participants was examined using Fisher’s exact test (Prism 5, Graph Pad).

## 3 RESULTS

### 3.1 Data at leave from work and at return to work in all participants

All participants completed the study and returned to work at 86.1 ± 108.3 days from the day of leave. Data at leave from work and at return to work in all participants (n = 30) are presented in Table 1. The mean dose of antidepressant taken by the participants was 15.8 ± 36.2 mg/day (fluvoxamine equivalent)^21^ at leave and 40.4 ± 37.2 mg/day at return. At return, the dose increased (*t* = 3.772, *P* < .001), and both SDS scores and STAI‐state scores decreased (*t* = 5.754, *P* < .001 and *t* = 6.246, *P* < .001, respectively), indicating that the psychiatric symptoms were ameliorated after receiving antidepressant treatment. Among the randomness scores, CB score improved (*t* = 2.577, *P* = .008), but IB and RNG scores remained unchanged (*P* > .05).

**Table 1:** HRV indices at leave and at return in all participants (n = 30)

	At Leave	At Return
	Mean (SD)	Mean (SD)
HR		
Rest (/min)	82.6 (12.1)	77.4 (9.6)^a^
Task/Rest	1.03 (0.06)	1.05 (0.06)
After/Rest	0.98 (0.05)	0.99 (0.03)
HF		
Rest (ms^2^)	89.4 (92.4)	155.5 (156.8)^a^
Task/Rest	1.27 (1.18)	0.77 (0.71)^a^
After/Rest	3.30 (4.33)	2.05 (1.74)
LF		
Rest (ms^2^)	270.5 (383.3)	569.4 (1546.6)
Task/Rest	2.59 (3.94)	2.07 (2.05)
After/Rest	2.72 (4.13)	2.61 (2.94)
LF/HF		
Rest	3.68 (3.72)	4.98 (10.55)
Task/Rest	2.36 (2.18)	3.28 (2.66)
After/Rest	1.26 (1.37)	1.99 (2.70)
		
CB	0.228 (0.137)	0.188 (0.102)^a^
IB	0.707 (0.197)	0.673 (0.200)
RNG	0.367 (0.079)	0.363 (0.070)
		
SDS	56.2 (8.6)	44.5 (9.6)^a^
STAI‐state	57.8 (10.6)	45.6 (12.9)^a^
Anti‐depressant	15.8 (36.2)	40.4 (37.2)^a^
(fluvoxamine‐equivalent, mg daily)		

Details of abbreviations are found in the text.

^a^ At Leave vs. At Return *P* <.05, paired t‐test.

At return, the HR Rest score decreased (*t* = 2.465, *P* = .010), HF Rest score increased (*t* = 2.224, *P* = .017), and HF Task/Rest ratio decreased (*t* = 1.921, *P* = .033). Other indices showed no changes at return (*P* > .05, Table 1).

### 3.2 Data in successful return and unsuccessful return groups

One month following their return to work, 19 patients continued their original work (SR group), while 11 failed to continue their original work (UR group). The data in the SR and UR groups are presented in Table 2. The mean age of the participants as well as the duration of sick leave did not differ between the two groups (*P* > .05). The numbers of men and women were 8 and 11 in the SR group, and 8 and 3 in the UR group, respectively. No statistical difference was observed between the two groups in terms of male‐to‐female ratio (*P* > .05).

**Table 2:** HRV indices at leave and at return in Successful and Unsuccessful Return groups

	Successful (n = 19)		Unsuccessful (n = 11)	
	At Leave	At Return	At Leave	At Return
	Mean (SD)	Mean (SD)	Mean (SD)	Mean (SD)
HR				
Rest (/min)	81.5 (14.1)	77.6 (10.7)	84.4 (7.7)	77.0 (7.7)^a^, ^a^
Task/Rest	1.03 (0.06)	1.05 (0.07)	1.03 (0.06)	1.04 (0.06)
After/Rest	0.97 (0.05)	0.98 (0.02)	1.00 (0.04)	0.99 (0.04)
HF				
Rest (ms^2^)	107.8 (106.7)	204.9 (174.7)^a^	57.7 (50.2)	70.0 (60.3)^b^
Task/Rest	1.29 (1.15)	0.36 (0.24)^a^	1.24 (1.28)	1.48 (0.70)^b^
After/Rest	3.25 (4.76)	1.38 (0.84)	3.38 (3.68)	3.22 (2.25)
LF				
Rest (ms^2^)	322.3 (451.5)	373.8 (284.0)	181.1 (212.8)	907.2 (2567.8)
Task/Rest	2.72 (4.67)	1.25 (1.18)	2.35 (2.41)	3.48 (2.50)
After/Rest	2.09 (1.88)	2.28 (1.87)	3.81 (6.41)	3.17 (4.27)
LF/HF				
Rest	3.53 (3.42)	2.38 (2.36)	3.95 (4.34)	9.48 (16.65)^b^
Task/Rest	2.13 (1.90)	3.59 (2.81)	2.76 (2.66)	2.76 (2.24)
After/Rest	1.40 (1.68)	2.04 (2.09)	1.01 (0.53)	1.91 (3.64)
				
CB	0.215 (0.156)	0.177 (0.113)	0.251 (0.100)	0.209 (0.078)
IB	0.718 (0.233)	0.653 (0.209)	0.690 (0.122)	0.708 (0.189)
RNG	0.370 (0.094)	0.360 (0.077)	0.363 (0.045)	0.368 (0.062)
				
SDS	56.2 (9.1)	40.7 (7.8)^a^	56.2 (8.1)	50.9 (9.2)^b^
STAI‐state	56.2 (10.6)	41.2 (10.8)^a^	60.6 (10.5)	53.3 (13.1)^a^, ^b^
Anti‐depressant	13.2 (35.5)	33.6 (34.9)^a^	20.5 (40.2)	52.3 (39.9)^a^
(fluvoxamine‐equivalent, mg daily)				
Age (years)	42.9 (11.3)		42.5 (14.0)	
Duration of leave (days)		90.8 (123.2)		78.0 (81.1)

Details of abbreviations are found in the text.

^a^ At Leave vs At Return *P* < .05, Bonferroni test.

^b^ Successful vs Unsuccessful *P* < .05, Bonferroni test.

At leave from work, no differences were observed in HR and HRV indices, task performance (CB, IB, and RNG), psychological scores (SDS and STAI‐state), and antidepressant dose (*P* > .05) between groups.

At return to work, HF Rest score increased (*F*(1, 28) = 4.30 for leave vs return effect, *t* = 3.036, *P* < .05), while HF Task/Rest ratio decreased (*F*(1, 28) = 8.05 for interaction, *t* = 3.710, *P* = .008) in the SR group. The UR group did not show such HF changes (*P* > .05). At return to work, the UR group showed significantly lower HF Rest score (*F*(1, 28) = 6.14 for SR vs UR effect, *P* = .020), higher HF Task/Rest ratio ((*F*(1,28) = 8.05 for interaction, *t* = 3.254, *P* < .01), and higher LF/HF Rest score (*F*(1,28) = 3.37 for SR vs UR effect, *t* = 2.452, *P* < .05) than the SR group.

The participants in both groups were taking increased dose of antidepressants (*F*(1,28) = 16.75 for leave vs return effect, SR group; *t* = 2.640, *P* < .05, UR group; *t* = 3.134, *P* < .01). The SR group exhibited a decrease in SDS score (*F*(1,28) = 31.70 for leave vs return effect, *t* = 6.929, *P* < .001). The STAI‐state score decreased in both groups (*F*(1,28) = 35.99 for leave vs return effect, SR group; *t* = 6.645, *P* < .001, UR group; *t* = 2.482, *P* < .05). At return, the UR group showed higher SDS and STAI‐state scores (*F*(1,28) = 7.63 for interaction, *t* = 3.151, *P* < .01 for SDS and *F*(1,28) = 4.72 for SR vs UR effect, *t* = 2.854, *P* < .05 for STAI‐state).

The randomness scores (CB, IB, and RNG) at leave from work were not significantly different from the scores at return to work in both groups (*P* > .05). The randomness scores of the UR group were not different from those of the SR groups both at leave and at return (*P* > .05).

## 4 DISCUSSION

The present study has clearly indicated that HRV indices in depressed patients at return to work from leave of absence due to illness are associated with the outcome of reinstatement. Unsuccessful reinstatement was associated with low HF Rest score, high HF Task/Rest ratio, and high LF/HF Rest score at return. These HRV conditions could be viewed as risk indices for unsuccessful return. At present, few objective measures are available to determine the appropriate time for reinstatement, and the HRV measurement can provide useful information regarding the feasibility of return to work.

HRV scores both at rest and at task are related to outcomes, indicating that not only the baseline autonomic conditions but the responses of autonomic system to mental load should be adequate in order to cope with various events after returning to work. The present method incorporating the mental task to autonomic measurement is useful because it simulates the actual situation at work.

The HRV changes related to unsuccessful return including low HF Rest score, high HF Task/Rest ratio, and high LF/HF were already reported in MDD patients.^9^ The results of the present study indicate that autonomic disturbances associated with depression were not ameliorated at return to work in the UR group. In the SR group, HF Rest score increased, while HF Task/Rest ratio decreased significantly, reaching the level of the healthy participants in our previous studies.^9^, ^10^, ^22^ The randomness scores of the task performance were not statistically different between the SR group and UR group as well as between at leave and at return. The present results were not due to the differences in task performance, but they were due to the changes in autonomic activity and reactivity. Amelioration of autonomic dysfunction revealed by HRV analysis could be related to psychopathological improvement. This finding suggests that the HRV indices are state dependent and can change by treatment; they can also serve as a biological marker reflecting the state of depressive illness. HRV measurement would help the psychiatrists to evaluate the depressive state not only when they are to judge the feasibility of return but also to evaluate the depressive symptoms in general.

The SDS scores were reduced in all UR patients although statistical significance was not reached in Bonferroni test. However, paired t test revealed significant reduction in SDS score when only the UR data were analyzed (*t* = 2.398, *P* = .018). STAI‐state score also reduced significantly in the UR group. In spite of these subjective recovery in addition to the remission of symptoms evaluated by the attending psychiatrist based on the DSM criteria,^11^ the patients in the UR group failed to return to the original work, indicating the difficulty to judge the feasibility of return by using the conventional psychopathological methods, although the SDS and STAI‐state scores were found higher retrospectively in the UR group at return. The self‐rating psychological scores are useful and should be used for assessment of return, but often convey inaccuracy due to subjective self‐evaluation. The decision of reinstatement depending on the conventional psychological method alone may be insufficient in some cases. Addition of the biological indices such as HRV should improve the assessment of reinstatement.

Some participants could adapt to work by changing the content of work or by utilizing the psychological and training programs.^6^, ^23^ These supports were not employed in our study patients, and the outcome of their reinstatement should be more directly related to their psychopathological condition upon resumption of work, which may involve the autonomic dysfunction. HRV measurements could be useful in assessing the risk of unsuccessful return in addition to the conventional psychiatric evaluation.

In the present study, HF is found to be related to the outcome of reinstatement. HF is known to reflect parasympathetic activity.^17^ Restoration of parasympathetic activity would be important to achieve the successful return to work. Low baseline HF may indicate unnecessary anxious state at rest.^22^ High LF/HF Rest score may reflect low baseline HF. High HF Task/Rest ratio could suggest that the parasympathetic activity is not switched off when the behavioral state is shifted from rest to task. Insufficient reactivity to workload in addition to baseline underactivity of parasympathetic system can be regarded as residual dysfunction related to unsuccessful return to work. The present study suggests that disturbance in the parasympathetic function is not only related to the pathophysiology of depression but to the ability of the patients to perform their daily activity including work.

The present study has some limitations. The study used a small sample size, and the outcome of return was evaluated only at 1 month after return to work. Hence, future studies with a larger sample size and with a longer period for evaluation of the return are warranted to determine other autonomic indices related to reinstatement. Other biological indices including brain circulation and skin conductance activity would also be interesting to investigate.

In summary, the present study has revealed that autonomic dysregulations in depressed patients at return to work from leave of absence due to illness are associated with the outcome of reinstatement. Unsuccessful reinstatement was associated with low HF Rest score, high HF Task/Rest ratio, and high LF/HF Rest score at return. These HRV conditions can serve as useful information for the assessment of the risk of unsuccessful return.

## CONFLICT OF INTEREST

None.

## AUTHOR CONTRIBUTIONS

TS involved in study concept and design, and drafting of the manuscript. TS, KM, YU, YA, HT, MT, RT, and MU involved in acquisition of data. TS, SS, MM, NK, SM, YO, HM, YT, YM, GM, TN, GS, TI, and TM involved in analysis and interpretation of data.

## DATA REPOSITORY

We cannot deposit the raw data in a public repository, since we did not obtain informed consent of the participants to make them publicly available. However, the data that support the findings of this study are available from the corresponding author on reasonable request.
